# Size Matters: Observed and Modeled Camouflage Response of European Cuttlefish (*Sepia officinalis*) to Different Substrate Patch Sizes during Movement

**DOI:** 10.3389/fphys.2016.00671

**Published:** 2017-01-17

**Authors:** Noam Josef, Igal Berenshtein, Meghan Rousseau, Gabriella Scata, Graziano Fiorito, Nadav Shashar

**Affiliations:** ^1^Mote Marine Laboratory, Directorate of Marine Biology and ConservationSarasota, FL, USA; ^2^Institute for Human and Machine CognitionPensacola, FL, USA; ^3^Eilat Campus, Department of Life Sciences, Ben-Gurion University of the NegevBeer Sheva, Israel; ^4^H. Steinitz Marine Biology Laboratory, Interuniversity Institute for Marine SciencesEilat, Israel; ^5^Stazione Zoologica Anton DohrnNapoli, Italy

**Keywords:** crypsis, cephalopods, vision, object size recognition, camouflage modeling, behavior, background matching, cognition

## Abstract

Camouflage is common throughout the phylogenetic tree and is largely used to minimize detection by predator or prey. Cephalopods, and in particular *Sepia officinalis* cuttlefish, are common models for camouflage studies. Predator avoidance behavior is particularly important in this group of soft-bodied animals that lack significant physical defenses. While previous studies have suggested that immobile cephalopods selectively camouflage to objects in their immediate surroundings, the camouflage characteristics of cuttlefish during movement are largely unknown. In a heterogenic environment, the visual background and substrate feature changes quickly as the animal swim across it, wherein substrate patch is a distinctive and high contrast patch of substrate in the animal's trajectory. In the current study, we examine the effect of substrate patch size on cuttlefish camouflage, and specifically the minimal size of an object for eliciting intensity matching response while moving. Our results indicated that substrate patch size has a positive effect on animal's reflectance change, and that the threshold patch size resulting in camouflage response falls between 10 and 19 cm (width). These observations suggest that the animal's length (7.2–12.3 cm mantle length in our case) serves as a possible threshold filter below which objects are considered irrelevant for camouflage, reducing the frequency of reflectance changes—which may lead to detection. Accordingly, we have constructed a computational model capturing the main features of the observed camouflaging behavior, provided for cephalopod camouflage during movement.

## Introduction

Animals often use camouflage to avoid detection by either predators or prey (Skelhorn and Rowe, 2016). Camouflage can take several forms: crypsis (avoiding detection) (Stevens and Merilaita, 2009), mimicry (resembling a defended organism) (Speed, 1993) and masquerading (resembling an inedible object) (Skelhorn et al., 2010). Crypsis in general, and background matching in particular, are examples of adaptation, where mismatch results in high susceptibility to detection (Ruxton et al., 2004; Caro, 2005a,b).

Coleoid cephalopods (octopuses, cuttlefish, and squid) are often preyed upon by marine mammals, eels, sharks and many other fishes (Aronson, 1991). Such selective forces drove this group of animals to develop various coloration capabilities and behaviors, including adaptive camouflage (Cott, 1940; Hanlon and Messenger, 1998; Barbosa et al., 2007). Adaptive camouflage is the capacity of animals to modify their appearance according to their habitat, to resemble specific background features in their immediate surroundings, or to perform background matching and context-dependent body patterning while moving (Keeble and Gamble, 1899; Gamble and Keeble, 1900; Josef et al., 2012, 2015; Jensen and Egnotovich, 2015). Cuttlefish can dynamically and rapidly camouflage themselves against a variety of natural backgrounds (Thomson, 1920; Hanlon and Messenger, 1998) using specialized tissues: the chromatophores, iridophores, leucophores, and papillae. These marine molluscs possess a keen visual system which can rapidly assess complex visual scenes and reflect them as camouflage body patterns, reviewed in Chiao et al. (2007). Body patterning, texture and body posture is adjusted to their intended audience (Boal et al., 2004) and is effected by background intensity (Chiao et al., 2007), spectrum (Akkaynak et al., 2013), contrast (Chiao et al., 2007, 2010), 3D environment structure (Buresch et al., 2011), background orientation (Barbosa et al., 2012) and object edges (Chiao et al., 2013). As in most evolutionary arms-races, the capacity to quickly alter one's body patterns and camouflage against visual backgrounds may have facilitated the development of visual mechanisms that enhance cephalopods' predators and prey ability to identify objects of interest; examples of such mechanisms include figure/ground discrimination by relative motion and edge detection (Land and Nilsson, 2012; Cronin et al., 2014). Previous studies have categorized cuttlefish's pattern repertoire to: uniform, mottled and disruptive (Hanlon and Messenger, 1998; Hanlon et al., 2007); this was recently implemented in an automated quantitative algorithm successfully classifying images of cuttlefish into these three categories (Orenstein et al., 2016). Coleoid camouflage capabilities have been intensively studied, yet little is known about how changes in appearance operate over variable timescales, or the mechanisms involved, ranging from short term reflectance change to longer phenotypic plasticity (Nettle and Bateson, 2015).

The benthic marine environments of coral reefs, sea grass or sandy seabed are constructed of many microhabitats largely characterized by a wide range of textures, brightness levels and contrast. Furthermore, flicker, or wave induced moving light patterns, also temporarily change the appearance of these backgrounds (Mcfarland and Loew, 1983). For a given cuttlefish swimming in such an environment, responding to small and possibly transient visual stimuli in its surroundings may subject the animal to dangerous mismatching, and the allocation of unnecessary processing effort. Hence, it is likely that the moving animal will react to patterns large enough to allow matching. Therefore, we hypothesize that a minimal threshold may exist for any Camouflage Eliciting Patch Size (CEPS). We conceive that this threshold represents the smallest background patch eliciting a quick dynamic camouflaging reaction. Note that “small” could be in terms of relative size, angular size, duration of encounter, or other terms relevant to the animal. Moreover, we hypothesize the existence of a positive correlation between patch-size and change in mantle reflectance.

In this study we tested the occurrence and intensity of the moving animal's reaction to visual patchs of various sizes. We identified the minimal CEPS threshold, and provide a possible camouflage model for a swimming *S. officinalis* cuttlefish.

## Materials and methods

In this set of experiments we were using the same experimental design and methodologies extensively detailed and described in Josef et al. (2015), modified mainly for the artificial backgrounds (see experimental design and testing procedure sections Experimental Design, Testing Procedure).

**Figure 1:**
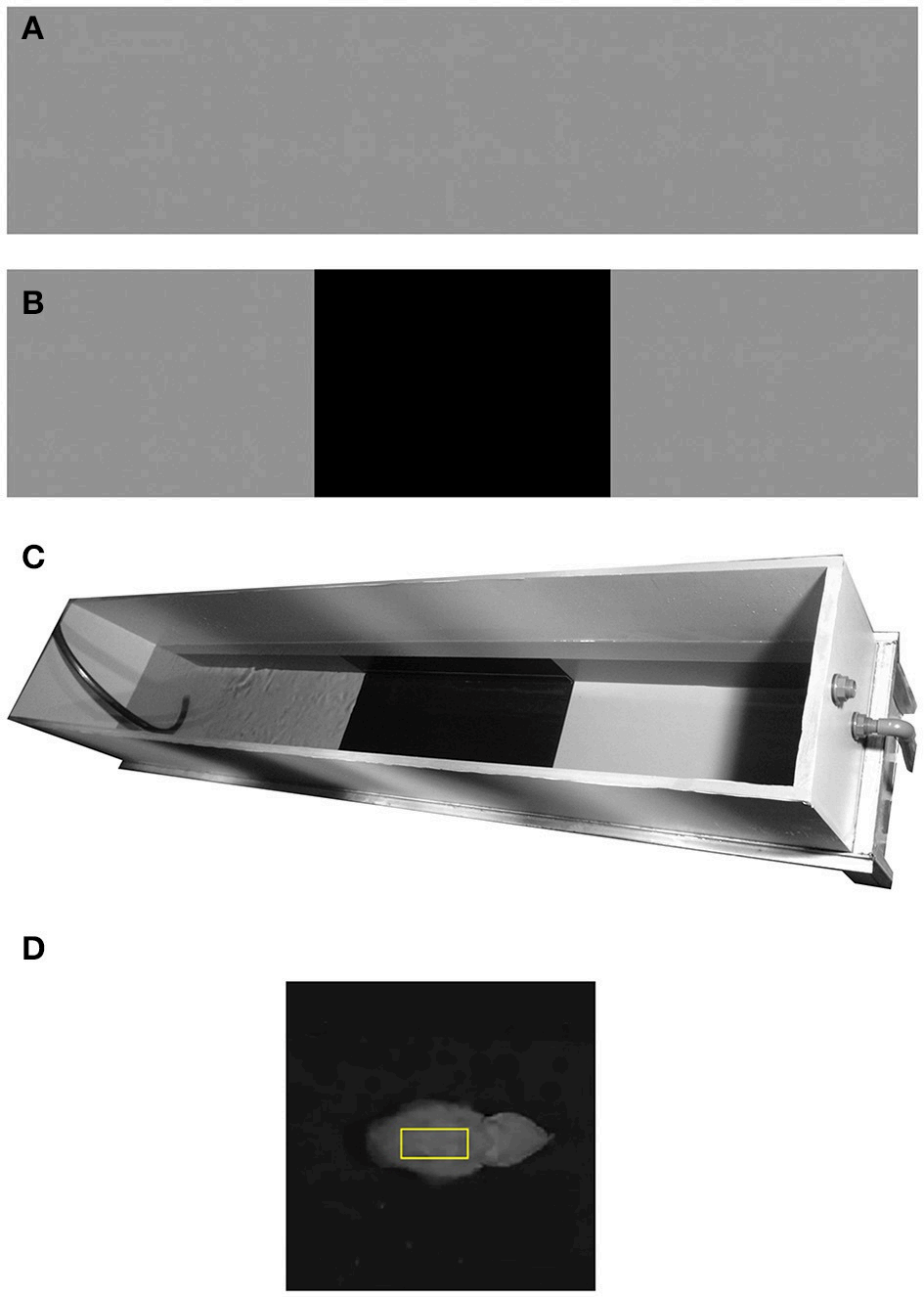
**Experimental set up. (A)** Control background –uniform 18% gray. **(B)** Dichromic background—providing a change in background reflectance. **(C)** Experimental tank with the dichromic pattern. **(D)** An example of a cuttlefish mantle with a 1000 pixel rectangular sample (yellow).

- Animals: Eight naive common European cuttlefish (*Sepia officinalis*), mantle length of 7.2–12.3 cm (10.2 ± 1.2 cm: mean ± SD) were collected from the Gulf of Naples, Italy and were held in separate tanks with running seawater, at the Stazione Zoologica Anton Dohrn in Italy, for 2 days of acclimatization. The cuttlefish were fed with live crabs, and maintained under a 12:12 (D: L) light regime. When experiments ended, all animals were returned to the Gulf of Naples. The experiments carried out in this study complied with the Italian National Legislation for animal experiments and with EU directive 2010/63 on the protection of animals used for scientific purposes (Smith et al., 2013; Fiorito et al., 2014).
- Experimental design: All visual cues and external stressors were minimized by performing the experiments in a secluded room with a curtain surrounding the set-up. An elongated tank (200 × 40 cm, water level 45 cm) was colored in a uniform 18% reflectance gray (Figure 1A); the reflectance throughout this study was based on a standard 18% gray card, photographed inside the elongated sea water tank, where 0 to 100% represents black and white respectively. All eight cuttlefish were placed in the elongated tank with either a control pattern (complete 18% reflectance gray; Figure 1A), or a dichromic pattern composed of three areas: 18% gray, 3% black, and 18% gray again (Figure 1B Each animal swam, one at a time, across the gray tank as a control, and over a set of six black patches of different sizes. The dark sections varied in length (3, 7, 10, 19, 29, 60 cm, all 40 cm in width spanning the width of the tank); these sections were added at the bottom center of the tank along the animals' swimming course (Figure 1C). Since cuttlefish preferentially respond to bottom rather than side stimuli (Taniguchi et al., 2015), the black patch covered the entire width of the tank but not the sides. The swimming cuttlefish were tracked and their mantle reflectance was continuously monitored. Since tactile information is a potential signal for camouflage, all textures were equally and completely smooth.
- Illumination across the tank was fairly homogeneous (350 ± 5 lux—measured with a PeakTech 5025 light meter) to avoid shaded areas or light reflections. The water in the experimental tank was replaced prior to each trial.
- Testing procedure: Animals were tested separately during the daytime (9:00–17:00). After being placed at one end of the experimental tank, each animal was left to settle for at least 5 min. We then waited until two conditions were met: (1) the animal remained motionless on one side of the tank; (2) the body color became uniform and generally matched the gray background, and remained stable for at least 2 min. The animals were then observed and video recorded as they moved in the tank, mostly crossing it along its length. If the animals did not move within 15 min of observation, they were motivated to cross the tank either by simply standing at one end of the tank, or by providing a shelter at the opposite side of the tank. Under no circumstances were the animals scared or strongly motivated, to minimize stress. In both control and dichromic conditions, animals were recorded crossing the tank, mantle first, from one side to the other (hereafter: “Full-cross”). Cuttlefish possess both anterior and posterior binocular visual fields which allow them to clearly see and plan their route while swimming forward or backwards (Watanuki et al., 2000). Thus, confining data acquisition to episodes of swimming mantle-first should not bias the results. In the control background, a full crossing of the tank provided information on the animals' changes in body color during motion over a constant background. Introducing the experimental dichromic backgrounds with the variably-sized black patchs allowed assessment of color changes as the animals swam over a gray-to-black and then a black-to-gray background transition. Patch widths were randomized and a single “full-crossing” over the control background and each of the six black patches were recorded for each of the eight animals. This protocol resulted in recordings of 48 experimental full crossings with 96 background transitions: 48 gray-to-black and 48 black-to-gray.
- Data acquisition: The animals movements were recorded using a SONY HDR-CX110 digital video camera mounted vertically above the tank providing a top-down view. To achieve high-resolution frames for analysis, the camera was set so its field of view covered the entire width and 70% of the tank's length- filming 1440 × 1080 pixels video files of 140 cm out of the 200 cm tank's length; the final 30 cm at each end of the elongated tank was not recorded.
- Data analysis: Cuttlefish possess a single, mid-wavelength visual pigment making them essentially color-blind (Marshall, 1996; Hanlon and Messenger, 1998; Mäthger et al., 2006). Moreover, most of the changes in the background and the cuttlefish display are monochromatic in nature, so we chose to look only at changes in reflectance and not in color. Therefore, only the green channel from all videos were gray-scale transformed, using the green channel alone. Videos were analyzed using a designated MATLAB™ code (Matlab version R2016a, MathWorks Inc., Natick, MA, USA). The code was utilized as follows: loading a video file, transforming each frame into a gray-scale intensity image, balancing each frame according to the 18% gray standard, manually tracking the animal in $\frac{1}{10}$ of a second intervals, and measuring the animal's mantle reflectance consisted of the average value of 1000 (40X25) pixels surrounding the center of the mantel (Figure 1D), velocity and relative position in relation to the next background. Although cuttlefish can present three types of body patterns—uniform (little or no variation in body pattern contrast), mottled (small or large-scale light or dark patches), or disruptive (non-repetitive high-contrast patches) (Cott, 1940; Hanlon and Messenger, 1998; Chiao et al., 2007), due to the uniform background in our setup, the animals always elicited a uniform body pattern in all cases. Therefore, we used the value of the mantle sample for data analysis. To characterize trends, we extracted and analyzed each section separately, paying special attention to the start and end points of each transition in body reflectance (Detailed methodology can be found in Josef et al., 2015). Then, the chromatic transitions start and end points were determined by manually selecting points that marked the beginning or end of change in reflectance. A start or end point was only chosen if the trend was maintained for at least three consecutive measurements. Once we set the beginning and end points of all transitions, we calculated the average slope, which represents the rate at which the animals match their backgrounds. Reflectance change variance was measured for each patch-size, quantifying the variance reaction between animals with patch size of increasing size. The Kruskal–Wallis one-way analysis of variance was used to compare the reflectance change percentage between the different patch sizes. This test allowed us to compare animal reaction to each pattern and to determine in which patch size the control and the test become significantly different.

Camouflage Sampling Area (CSA) is an important region we have defined as a partially occluded oval sub-sample of the environment relevant for the animal's camouflage and visually sampled for dynamically matching it. In our purposed model, the cuttlefish modifies its mantle reflectance according to the mean reflectance captured by its oval field of view.

**Figure 2:**
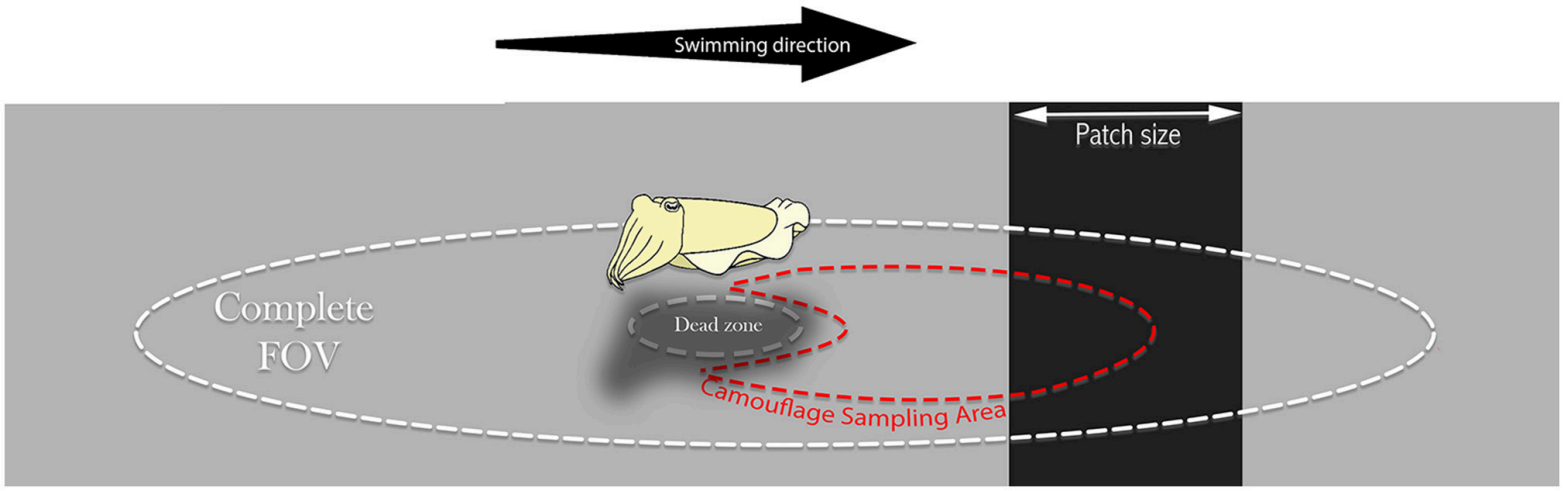
**Relevant Cuttlefish's fields of view**. The oval cuttlefish Complete Field of View (FOV) is restricted only by its optic and physiological features. We suggest a Camouflaging Sampling Area (CSA) as the relevant sampling area, sized and averaged by the observing animal to match its mantle reflectance under certain conditions for background matching purposes. The CSA is likely to be skewed forward to allow anticipation. Both fields are affected by the visual dead-zone under the animal's body.

**Figure 3:**
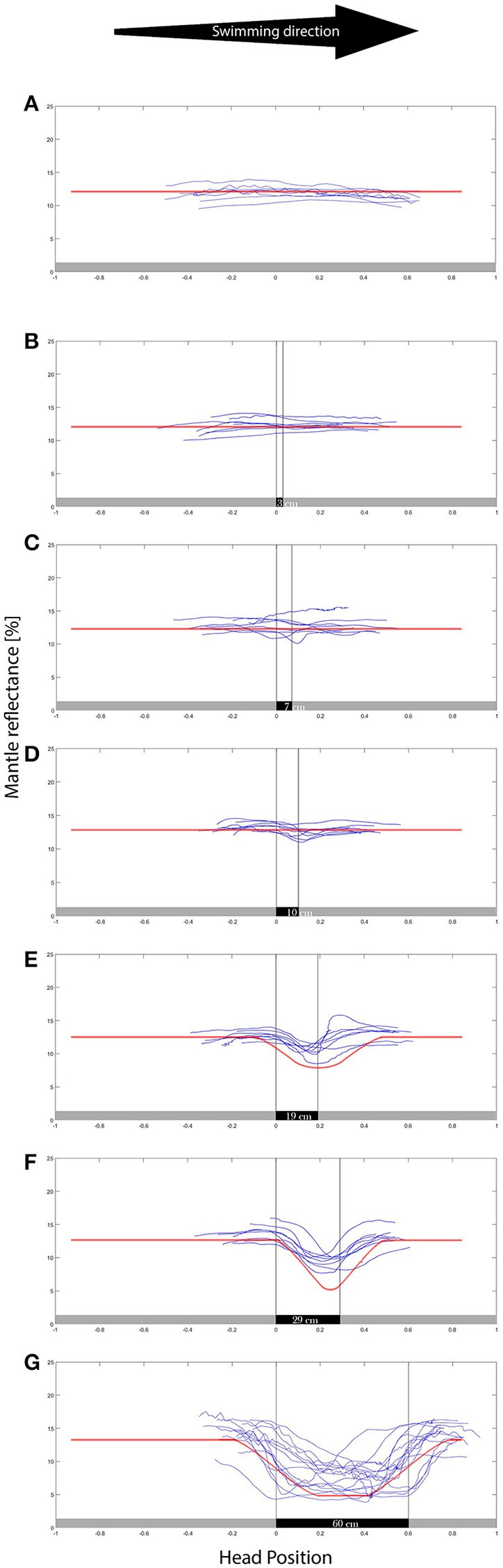
**Mantle reflectance values while swimming in the direction indicated over the different backgrounds** (**A–G** representing 0–60 cm patches respectively). The X axis is the position of the head/eyes of the cuttlefish, where the 0 represents the transition between gray and the black patch. The animals showed little response to the smaller (0–10 cm) group of patches, exhibiting a growing reflectance-matching behavior to the wider (19–60 cm) background patches. *N* = 8. The red lines are superimposed over the data, representing the computer model results. The model well represents the animal camouflaging behavior.

(g) Modeling the Field Of View (FOV) and the animal's behavior**:** We used MATLAB™ (Matlab version R2016a, MathWorks Inc., Natick, MA, USA) image analysis tool to simulate a dynamic environment where we sampled the changing background (emulating a swimming cuttlefish) (Figure 2). Using a designated MATLAB code, we performed the following:
  1. We created a virtual black and gray arena simulating the experimental tank, with increasing in size black patches.
  2. We then created an oval sampling area (4530 pixels), which is analogous to the cuttlefish' oval field of view. The algorithm then computes the average reflectance across all pixels within that oval sample, and records it. Sampling began at the bright area (18% reflectance gray), moving one pixel at a time across the virtual arena toward the black patch (3% reflectance black), and out to the bright area. The model graphs were then superimposed over the actual results graphs (Figure 3).
  3. Virtual mantle: We created a virtual mantel (rectangle 40X25 pixels) with a dynamic reflectance, which is modified (under certain circumstances) according to the mean reflectance, captured in the oval Camouflaging Sampling Area (CSA). Moving one pixel at a time, the sampled averages were combined with a stochastic behavior factor (±3%), reflecting behavior variance between individual animals. The full conditioning of the model is described in the discussion part and **Figure 5**.

## Results

As expected, while swimming in the uniform gray control tank, all eight animals maintained their overall light and uniform body coloration, matching the background throughout their movement (Figure 3A). As patch size increased we observed a gradual increase in number of animals that elicited a camouflage response with increasing patch size and a notable increase in intensity change (darkness level when over the black patch) was recorded for background patches ≥10 cm in length (Figure 3).

**Figure 4:**
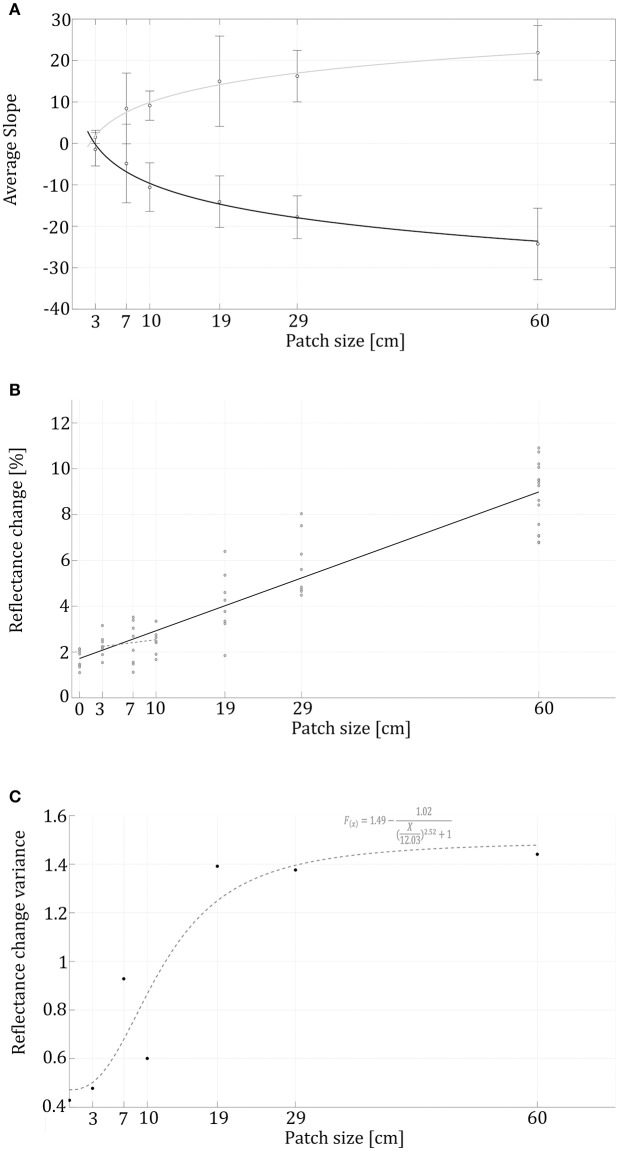
**Reflectance change rates and variance in correlation to patch sizes. (A)** Logarithmic black trendline represents swimming into a patch (*R*^2^ = 0.96), while the gray trendline relates to the animals swimming out of the patch onto the gray background (*R*^2^ = 0.95). Reflectance change rate (slopes) validates a symmetrical behavior while swimming into the patch and out of it. **(B)** The black linear trendline (*f*_(*x*)_ = 0.12*X* + 1.72, *R*^2^ = 0.87) represents the reflectance's correspondence to the patch sizes (*R*^2^ = 0.87). **(C)** The variation in reflectance change within each treatment with a sigmoidal dashed trend line (*R*^2^ = 0.88).

Comparing animals' reflectance in crossings from a gray to a black background vs. crossings from a black to gray background did not show any significant differences. For example, the trend lines' slopes of animals going onto the patch and going out toward the gray background shows high symmetry around the x axis and both follow a logarithmic trend (Figure 4A; *R*^2^ = 0.96, *R*^2^ = 0.95, respectively). Therefore, from here on, we will only address reflectance change behavior without distinguishing between whether it was from gray to black or from black to gray.

The entire cuttlefish's mantle changed simultaneously, without notable differences between posterior and anterior similar to the findings of a previous study (Josef et al., 2015).

In the control group, without visual background change, animals presented an average change in reflectance of 1.69 ± 0.40 %, while 4.02 ± 2.9% in the treatment group. The reflectance change had a positive linear correlation to patch-size (*r* = 0.93, *p* < 0.01), demonstrating that swimming over increasingly large black patches caused the animals to change their appearance faster (Figure 4A), and the overall reflectance-change increased with high correlation to patch size (Figure 4B). The Kruskal–Wallis test conducted on the seven different conditions (control and six patch sizes) validated a significant difference (χ^2^ = 42, df = 6, *n* = 8, *p* < 0.001) between the control and the 19, 29, and 60 size patches. As patch size increased, the first patch evoking a significantly different reaction than the control was the 19 cm patch.

When looking at the change rate (measured as the sigmoidal reflectance slopes), most animals behaved symmetrically while swimming into the patch and out of it. For both cases, logarithmic curve fit best describes the relationship between the average curve slope and the patch-size (*R*^2^ = 0.98 and *R*^2^ = 0.989 respectively). Symmetric behavior also expressed in a similar interception point with the x axis (2.25 and 2.897 respectively). The sigmoidal trend line (*R*^2^ = 0.88) shows a lag in reflectance-change variance (Figure 4C). Note is that Figure 4A represents the cuttlefish's reflectance change-rate without addressing the magnitude of the change.

Weak but significant correlation was found between the extent of reflectance change and the animal's average swimming velocity (*r* = 0.26, *p* < 0.05) throughout the different patch sizes (See Supplementary Figure 1).

## Discussion

To remain cryptic, it is essential for a moving cuttlefish to continuously adjust its appearance according to its changing background. Josef et al. (2015) showed that these animals can also anticipate and match upcoming backgrounds resulting in a gradual, sigmoidal-like function of background matching while moving. Previous studies identified and categorized specific background features that could elicit different skin patterns (Hanlon and Messenger, 1998; Chiao et al., 2007, 2010; Barbosa et al., 2008).

A welter of shapes, brightness levels, and textures, constantly stimulates the visual system of these animals, creating an enormous amount of information. At any given moment, this information must be reduced and prioritized, to obtain a comprehensive image with minimal use of data processing and memory. In primates, for example, visual recognition of objects depends on the transmission of information from the striate cortex through pre-striate areas into the inferior temporal cortex (Ozaki et al., 1983). The ability to filter out irrelevant visual information is required in developing attention and addressing the most relevant cues in any given visual scene. Moreover, the filtering of irrelevant information from the receptive fields underlies the ability to identify and remember the properties of a particular object out of the many that may be represented (Moran and Desimone, 1985). In the context of camouflage, such mechanisms would be beneficial for a static animal selecting a relevant object/background to match, as well as for a moving animal screening irrelevant cues as they appear.

There are two basic aspects that constrain visual attention. The first is the limited capacity for processing information. At any given time, only a small amount of the available information can be processed and used in the control of behavior. The second is selectivity—much of the information available is not relevant to the animal's tasks and hence animals need to filter out redundant or irrelevant information.

The artificial uniform backgrounds provided a simplified visual environment in which the camouflage of a swimming cuttlefish could be examined and modeled with regard to the patch-size encountered. Here, we would like to stress that in the wild, a clear step like transition between two uniform backgrounds is rare, as most natural scenes include a blending phase comprising complex backgrounds affecting each other. Although very interesting and highly important for further understanding of this process, in the current study we tried to model one of the simplest camouflaging feature and did not study transitions between mottled or disruptive patterns. Nonetheless, this apparatus simplified the visual field and minimized the behavioral response to a single type of reflectance change without addressing complicating factors such as patterns, textures and others.

Animals responded to the size of the patches in the background, yielding stronger changes of reflectance as the patch size increased; camouflage responses occurred more rapidly and were seen in more animals as they swam over larger patches (Figure 3), while both intensity and rate of reflectance change increased accordingly (Figure 4). Although it is hard to decipher what underlies the variation between the control and the experimental patches for patches up to 10 cm in width, a noticeable difference in rates and in reflectance magnitude was found between the 10 cm patch and the following 19 cm patch size. This difference means that the animals reacted significantly more strongly than to a 19 cm (and wider) patches than to the first three (0–10 cm) patches. This is the first evidence for the existence of CEPS in moving cephalopods suggesting that a possible CEPS threshold laying somewhere between these values. It is worth noting that Chiao (Chiao and Hanlon, 2001) and Hanlon and Zylinski et al. (Zylinski et al., 2011) alluded to a CEPS in stationary cuttlefish.

The first three Patches (3, 7, and 10 cm) elicited a mild change in mantle reflectance. The larger patch sizes (19, 29, and 60 cm) elicited a much more noticeable change in reflectance (both visually and numerically) making a faster transition with a positive linear correlation to patch size. The results support the existence of a CEPS threshold somewhere between the 10 and the 19 cm patch width, for average animal mantle lengths of 10.2 ± 1.2. Hence, the suggested CEPS threshold is slightly larger than the animal's mantle length.

In addition to the basal variation seen in the control group, we found a positive correlation between patch size and variance in the animals' reflectance change; this change is represented by a sigmoidal function. This sigmoidal fit (Figure 4C) also demonstrates that the greatest increase in reflectance variance took place for the 10–19 cm patch size range. The limited variances within the smaller-sized patches combined with the moderate reflectance change suggests a subtle behavioral reaction to smaller patch sizes; while the larger patches induced a stronger behavioral response, followed by larger reflectance variance. This serves as additional supportive evidence for a greater reaction to the larger patch sizes and a CEPS in the range of the animal's body size (in our case patch sizes somewhere between 10 and 19 cm).

It is conceivable that optic flow might have influenced visual perception and decision-making as our test animals swam between backgrounds. In our experimental trials, the animals swam rather gently without jetting; optic flow likely did not change during these relatively slow swim rates. However, when observed velocities did change, we saw no obvious relationship between the cuttlefishes' velocities and reflectance change. These observations support our conclusion that the visual cue of dimension/magnitude is a primary driver of the animals' reflectance change.

Matching very small background patches and paying attention to many details in it requires a large amount of attention and processing effort. Such a delicate process, is highly sensitive to errors and inconsistencies—inevitably causing the cuttlefish to be conspicuous. The lack of response to small patches might act in according to fitness considerations such that, individuals that changed their reflectance to very small landscape features may have an excess energy investment in camouflaging, or possibly that erratic change in coloration ultimately made them more conspicuous.

However, from the results, it seems there is a very weak reaction to the presence of these objects or patches. We therefore suggest that the cuttlefish preforms patch size estimation with a body-size CEPS threshold. If the threshold is not met, the cuttlefish expresses no change in mantle reflectance, whereas above this threshold they average a partially-occluded oval shaped CSA (Watanuki et al., 2000), resulting in a sigmoidal change in the sample model. This matches all observations in our previous work (Josef et al., 2015). Moreover, according to Josef et al. (2015), cuttlefish show an anticipation behavior, which might be explained if the CSA is in the direction of the animal's movement (Figure 2). Considering what is already known regarding cuttlefish background intensity matching (Chiao et al., 2007; Buresch et al., 2015), and the computer model results—we propose that these animals average an approximate oval-shaped subsample of the substrate in the direction of their movement. Such an averaging may also explain the moderate change in some animals in the presence of the small patches and increased reaction when larger patches are introduced. In the 3, 7, and 10 cm patches, some animals responded with a moderate reflectance change while others did not respond. If the animals had been continuously averaging a CSA and changing their mantle appearance accordingly, we would expect to see an increasing reaction throughout—even in the small patches. Since this did not happen, we suggest that a visual evaluation process is involved before the animals cross to the next background. This also corresponds with the findings of Josef et al. (2012) who showed a selective process in octopuses camouflage responses. On the other hand, the sigmoidal reaction signifies that the animals do not use an average reflectance value as the only threshold—which would result in a step function (see Supplementary Figure 2). Furthermore, the fact that in the 3,10, and 19 cm patches, 0, 50, and 100% of the animals respectively elicited a camouflage response indicate individual behavioral variation.

**Figure 5:**
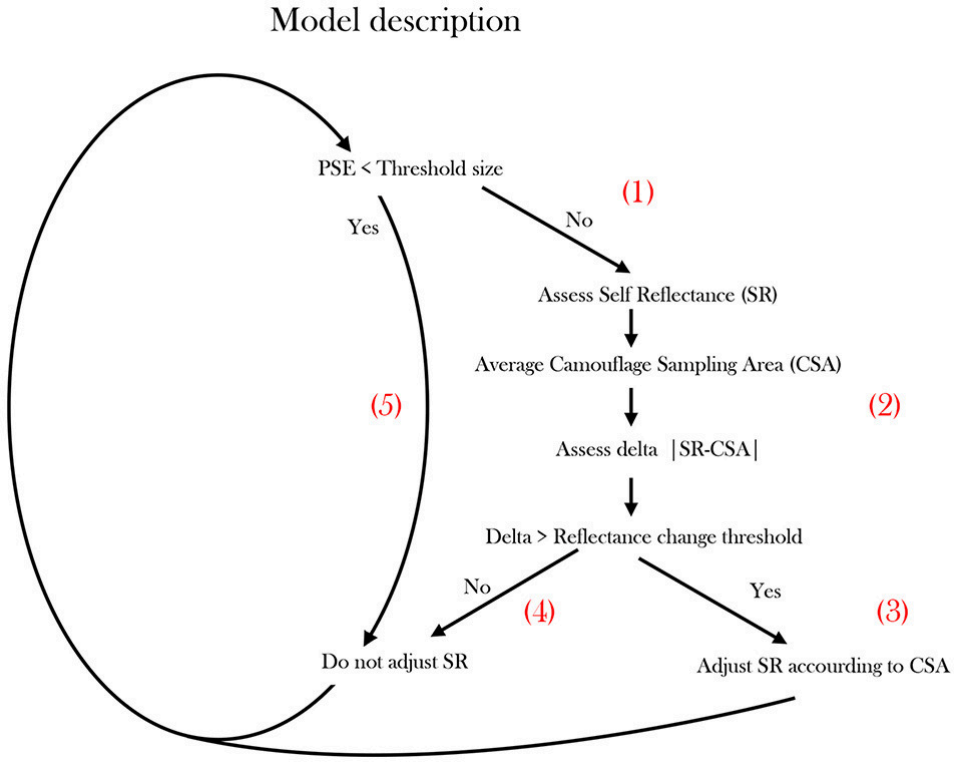
**Proposed operational model describing cuttlefish Camouflage behavior**. The model scheme simplifies the camouflaging process undertaken by a swimming cuttlefish when encountering different background cues. Patch Size Estimation (PSE).

In conclusion, based on previous as well as our current studies, we propose a new camouflage behavioral model for a swimming cuttlefish (Figure 5). We believe that two relevant oval visual fields: (1) the Entire Field Of View (the complete field of view of the animal, restricted only by its optic limits), and (2) the CSA (an oval subsample, skewed to the direction of animal movement: Figure 2) are primarily operative in cuttlefish camouflage responses to visual stimuli. We believe that the model might operate as follows.

The cuttlefish preforms Patch Size Estimation (PSE), possibly using depth perception visual cues (Josef et al., 2014), optic flow (Sun et al., 2014) or possibly by combining multiple visual cues. Specifically, it constantly scans for patches smaller than the CEPS threshold (segment 1, Figure 5). As long as a patch is not identified, the cuttlefish gauges whether its Self Reflectance (SR) is significantly different from the background. The latter distinction requires the cuttlefish to assess its own Self-Reflectance (SR) and averaging an approximate oval Camouflaging Sampling Area (CSA). Then it calculates the delta between its immediate background to its own reflectance |SR-CSA|—evaluating its current cryptic status (segment 2, Figure 5). If the reflectance difference is larger than a threshold, the cuttlefish modifies its reflectance according to the CSA (segment 3 in Figure 5). If the threshold is not surpassed, no change in reflectance will be elicited (segment 4 in Figure 5). In cases where the PSE is smaller than the CEPS threshold (body-size in our case), no change in reflectance will be elicited (segment 5, Figure 5).

Cuttlefish are well known for their dynamic, responsive and rapidly adjusting camouflage patterns and background matching. In the current study we did not experimentally confirm the existence of a reflectance delta threshold, yet a self-reflectance awareness and crypsis assessment are clearly required for such responses (Figure 5, segment 2).

Although our suggested model captures the main features of the cuttlefish camouflage, it is likely that the mechanism is more complex. For example, the averaging of a CSA might be a weighted average, with weight higher at the center of the shape; or a missing-oval shape of the CSA might be non-symmetric and skewed forward. More studies—especially using various artificial and natural patterns—will be required to further compare cuttlefish camouflage with our conceptual model performance and to refine the model accordingly. Finally, this model provides a first step in applying cephalopod camouflage in the growing field of biomimicry, allowing the implementation of the observed camouflage behavior in machine learning protocols, dynamically camouflaging protocols for both recreational and defense purposes.

## Author contributions

Conceptual contribution and study design: NJ, GS, and NS. Acquisition of data: NJ, NS, and GF. Analysis and interpretation of data: NJ, NS, IB, and MR. Drafting of manuscript: NJ, IB, GF, and NS. Critical revision: NJ, IB, MR, GS, GF, and NS.

## Funding

NJ and NS received financial support from the European Community for the access provided to Stazione Zoologica in Naples, through the ASSEMBLE program [EC/FP7 Grant Agreement No. 227799]. The funders played no part in the design of the study, data collection and analysis, decision to publish, or preparation of the manuscript.

### Conflict of interest statement

The authors declare that the research was conducted in the absence of any commercial or financial relationships that could be construed as a potential conflict of interest.

## Supplementary material

Supplementary Figure 1 **Animal's velocity had no effect on the reflectance change values**. We found no correlation between the two variables (correlation coefficient is 0.26).

Supplementary Figure 2 **From the model we learn that if the animals were continually and indiscriminately averaging a sampling area (CSA) while only responding to a reflectance threshold, a step-function in reflectance would emerge**. Such a sudden change in reflectance would create a drastic change in the animal appearance, in striking contrast to the results of the current and previous studies. Therefore, we conclude that the animals do not average the CSA indiscriminately and continuously, but they instead decide whether to camouflage (or not) in response to the upcoming patch on approach and not upon arrival. In this manuscript we show that the CEPS could well support this response by offering a selective threshold.
