# Supplementary figures and images for: Size Matters: Observed and Modeled Camouflage Response of European Cuttlefish (Sepia officinalis) to Different Substrate Patch Sizes during Movement
Source: Front Physiol. 2017 Jan 17;7:671. doi: 10.3389/fphys.2016.00671 (PMC5239790; doi:10.3389/fphys.2016.00671)

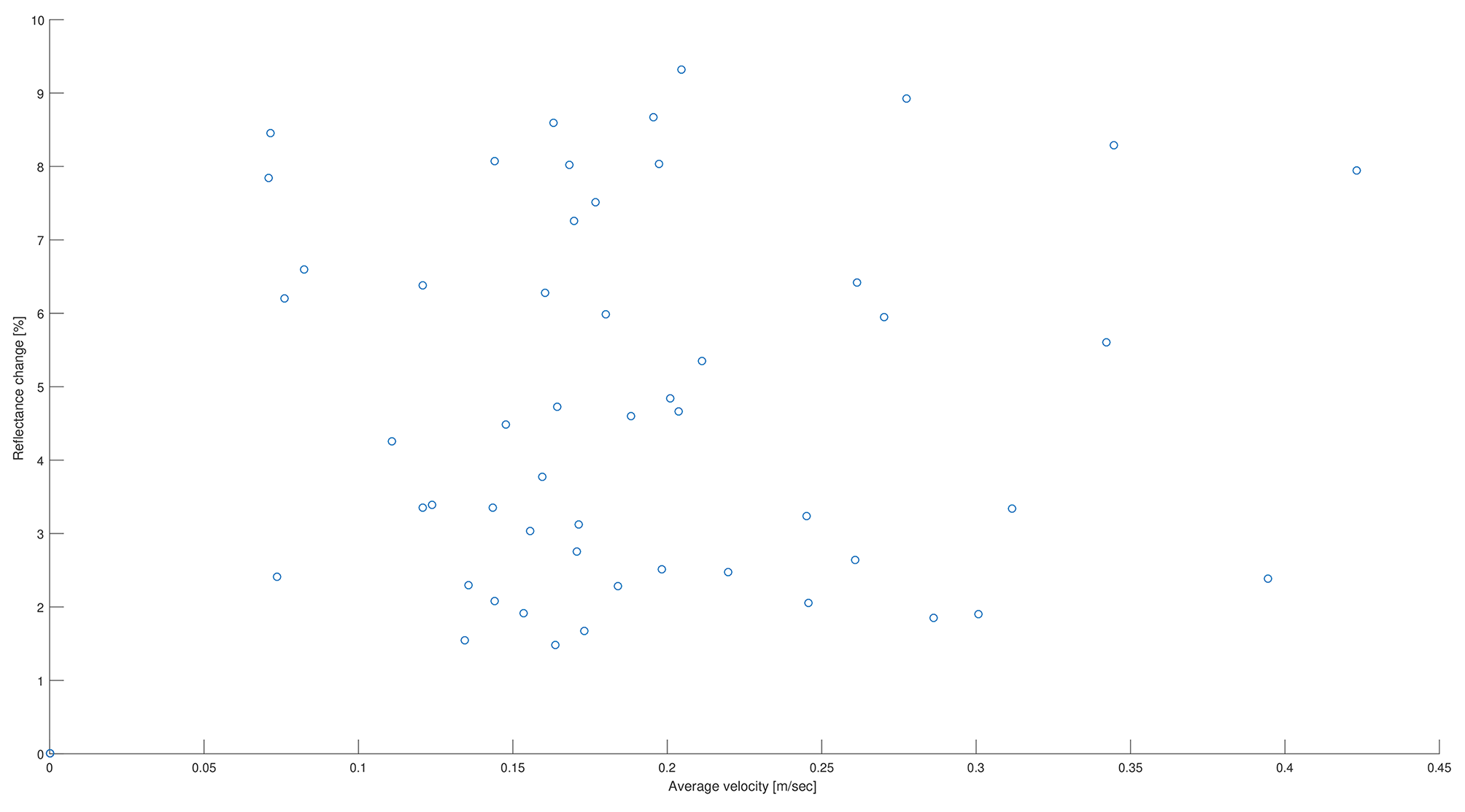

Supplement: Supplementary Figure 1 — Animal's velocity had no effect on the reflectance change values. We found no correlation between the two variables (correlation coefficient is 0.26). [file Image1.TIFF]

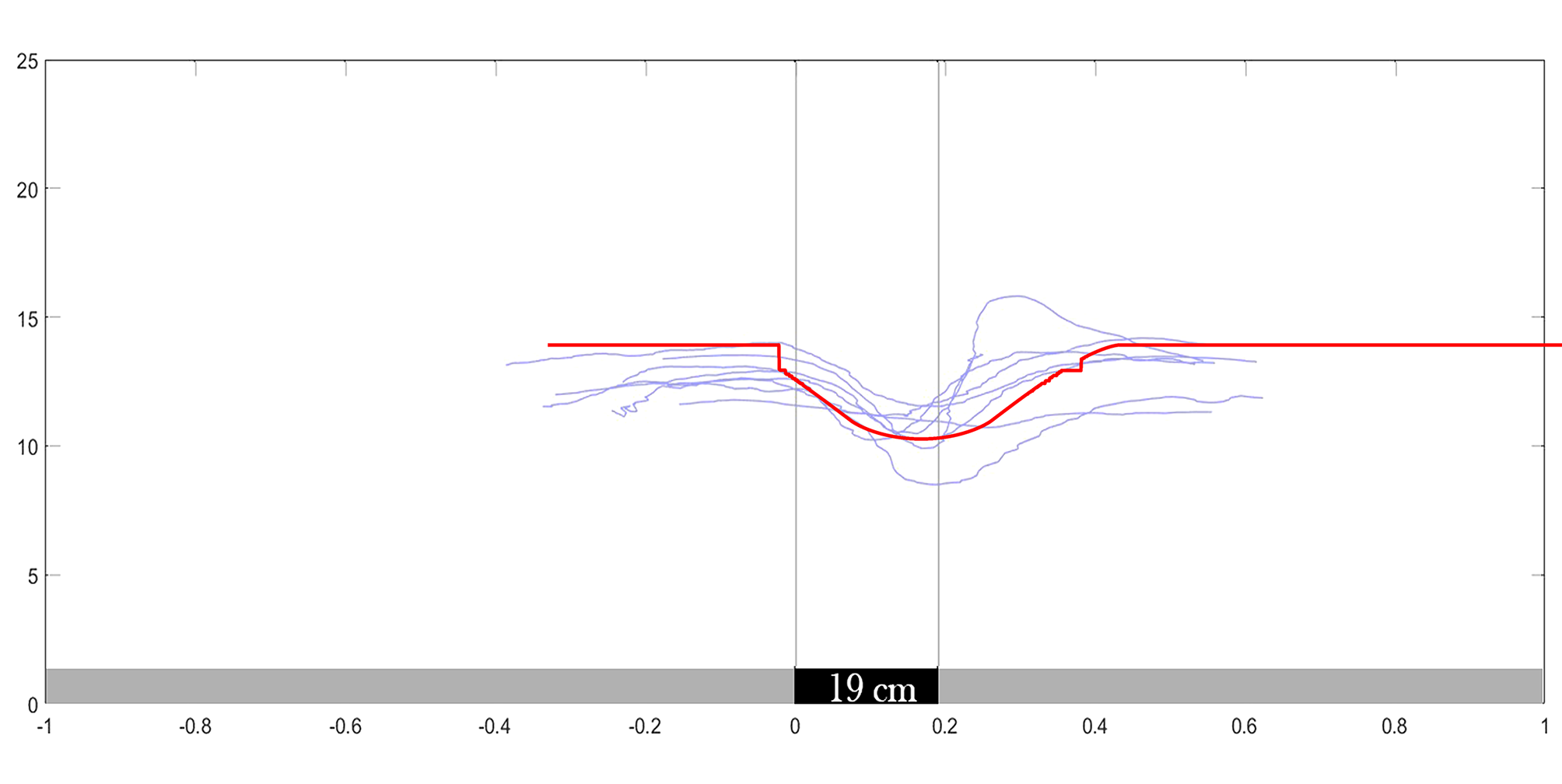

Supplement: Supplementary Figure 2 — From the model we learn that if the animals were continually and indiscriminately averaging a sampling area (CSA) while only responding to a reflectance threshold, a step-function in reflectance would emerge. Such a sudden change in reflectance would create a drastic change in the animal appearance, in striking contrast to the results of the current and previous studies. Therefore, we conclude that the animals do not average the CSA indiscriminately and continuously, but they instead decide whether to camouflage (or not) in response to the upcoming patch on approach and not upon arrival. In this manuscript we show that the CEPS could well support this response by offering a selective threshold. [file Image2.TIFF]
